# Cancer-specific utility: clinical validation of the EORTC QLU-C10D in patients with glioblastoma

**DOI:** 10.1007/s10198-024-01729-4

**Published:** 2024-11-20

**Authors:** Simone Seyringer, Micha J. Pilz, Andrew Bottomley, Madeleine T. King, Richard Norman, Eva M. Gamper, EORTC Quality of Life Group

**Affiliations:** 1 Department for Psychiatry, Psychotherapy and Psychosomatic Medicine,University Hospital of Psychiatry II, Medical University of Innsbruck, Innsbruck, Austria; 2 Department of Social Psychology, Personnel Development and Adult Education, Johannes Kepler University Linz, Linz, Austria; 3 School of Psychology, University of Sydney, Camperdown, NSW Australia; 4 School of Population Health, Curtin University, Perth, WA Australia; 5 Department of Nuclear Medicine, Medical University of Innsbruck, 6020 Innsbruck, Austria; 6 European Organisation for Research and Treatment of Cancer, Brussels, Belgium

**Keywords:** Glioblastoma, Health-related quality of life, QLU-C10D, EQ-5D-3L, Validity, Utility, D61 allocative efficiency, Cost–benefit analysis

## Abstract

**Introduction:**

Many health economic evaluations rely on the validity of the utility measurement for health-related quality of life (HRQoL). While generic utility measures perform well in HRQoL assessments of many diseases and patient populations, appropriateness for cancer-specific disease burdens needs attention and condition-specific measures could be a viable option. This study assessed the clinical validity of the cancer-specific EORTC QLU-C10D, a utility scoring algorithm for the EORTC QLQ-C30, in patients with glioblastoma. We expect the EORTC QLU-C10D to be sensitive and responsive in glioblastoma patients. Furthermore, we compared its statistical efficiency with the generic utility measure EQ-5D-3L.

**Methods:**

We used data from a multi-center randomized controlled trial (NCT00689221) with patients from 146 study sites in 25 countries. Both, the QLQ-C30 and the EQ-5D-3L, had been administered at seven assessment points together. Utilities of both measures were calculated for four country value set (Australia, Canada, UK, USA). Ceiling effects, agreement (Bland–Altman plots (BA), intra-class correlation (ICC)), were calculated to analyze construct validity. Sensitivity to known-groups (performance status; global health) and responsiveness to changes (progressive vs. non-progressive; stable vs. improved or deteriorated HRQoL) were investigated for clinical validity. Relative Efficiency (RE) was calculated to compare statistical efficiency of both utility measures.

**Results:**

435 patients were included at baseline and six subsequent time points (median timeframe 497 days). QLU-C10D country value set showed negligible ceiling effects (< 6.7%) and high agreement with EQ-5D-3L (ICC > 0.750). BA indicated that differences between both utility measures increased with deteriorating health states. While the QLU-C10D was more sensitive to global health groups (RE > 1.2), the EQ-5D-3L was more sensitive to performance status groups (RE < 0.7) than the other utility measure. Statistical efficiency to detect differences between change groups and within HRQoL deterioration group (RE > 1.4) favored QLU-C10D in 18 of 24 (75%) and 20 of 24 (83%) comparisons with the EQ-5D-3L respectively. Responsiveness to overall HRQoL change (RE > 3.4) also favored the QLU-C10D.

**Conclusion:**

Our results indicate that the QLU-C10D is a valid utility measure to assess HRQoL in patients with glioblastoma. This facilitates the investigation of HRQoL profiles and utilities in this patient population by administering a single questionnaire, the EORTC QLQ-C30. Efficiency analyses point to higher statistical power of the QLU-C10D compared to the EQ-5D-3L.

**Supplementary Information:**

The online version contains supplementary material available at 10.1007/s10198-024-01729-4.

## Introduction

Patients diagnosed with glioblastoma are confronted with an incurable disease and the outlook of a markedly limited life span with considerable health deterioration [1]. Frequently, patients already show an impaired health state at diagnosis and a wide range of symptoms, some of which are not common cancer symptoms [2]. During the diagnostic phase they frequently suffer from cognitive deficits, seizures and headaches. As the disease progresses, nausea, vomiting, fatigue, drowsiness, aphasia and motor deficits often start to appear [3, 4]. To evaluate impact of disease or treatment, measures are needed which adequately capture patients’ experience [5].

There are well validated HRQoL instruments available for brain tumor patients, such as the European Organisation for Research and Treatment of Cancer (EORTC) Quality of Life Core Questionnaire QLQ-C30, along with its brain tumor-specific instrument, the QLQ-BN20 [6–9]. These are most frequently used as secondary outcome measures in cancer clinical trials in this field [10]. When it comes to health economics and cost-utility studies though, there is a lack of disease-specific instruments. Analysis in these contexts rely commonly on generic measures, to facilitate comparisons across diseases. Further, health economic evaluations are based on a different measurement approach than HRQoL profile measures, like those mentioned above. Health states for health-economic evaluations are represented by utilities, numeric values of a cardinal scale.

Utility instruments are designed to take into account the preference towards a particular health state over another to value health states [11]. They consist of a health classification system—a set of HRQoL aspects, which may take on different severity levels—and a preference-based scoring algorithm. Thereby individual patient responses are converted into one utility value, that expresses the value which has been assigned to a particular health state by the general population [12]. Utility scales are anchored at 0 (representing being dead) and 1 (full health). Negative values are possible at country-specific value sets, representing health states worse than being dead [13, 14].

A frequently used HRQoL utility measure in health economic assessments, the EQ-5D-3L, is a well validated generic measure for a broad range of diseases and populations [15, 16]. This measure captures five dimensions on generic health aspects each with three response options. So, this measure is able to describe 3^5^ = 243 distinct health states. While in general the EQ-5D-3L performs well in a cancer setting, limitations in capturing cancer symptoms like fatigue or nausea, have been discussed [16–18]. Improvements of the updated version, the EQ-5D-5L with five response options for each domain, have been demonstrated, but a consensual English value set is not available yet and it is not clear whether the expansion of response categories is sufficient to resolve described short comings or if disease-specific domains still may add valuable information ([19–21]).

The QLU-C10D is a more recently developed scoring algorithm for the QLQ-C30, using 10 dimensions with four severity levels (i.e. theoretically describing 4^10^ = 1,048,576 distinct health states) that allows researchers to obtain utility scores from QLQ-C30 data [22, 23]. Therefore, the QLU-C10D is expected to capture a greater range of health states relevant to cancer patients and thus to be of potential added value to the measurement of utilities in cancer patients.

The primary objective of this retrospective study was to assess the clinical validity of the QLU-C10D in patients with newly diagnosed glioblastoma. Clinical validity is a psychometric requirement of patient-reported outcome measures, proofing that the instrument is able to detect clinically relevant differences and changes [12, 18]. Our study therefore adds psychometric information to the QLU-C10D portfolio complying with COSMIN standards on quality criteria for patient-reported outcome measures [24]. Additionally, we compared the QLU-C10D with the generic EQ-5D-3L with regard to statistical measurement efficiency. Both measures were administered in the course of the original study at each assessment. By using and comparing value sets from four different countries sharing the same language, namely for Australia (AUS), Canada (CAN), United Kingdom (UK) and USA, we furthermore provide an insight into potential cultural differences of the QLU-C10D utility weights, unbiased by differences that may result from translations. These regions represent a large proportion of the nationalities in the original study sample.

## Methods

### Original data source

Data for this analysis was drawn from an EORTC cancer clinical trial (ClinicalTrials.gov, NCT00689221), a global, multi-center, randomized, open-label, phase 3 trial. 545 eligible newly diagnosed glioblastoma patients with a methylated MGMT promoter, were randomly assigned to treatment arms: either treated with temozolomide chemoradiotherapy alone (TMZ) or with added Cilengitide [25]. Median follow-up time was 29 months. The present analysis encompasses baseline and six assessments thereafter (T1–T6), including follow-up at six months after treatment.

Patient data with completed HRQoL measures at baseline was used for the current analysis. For responsiveness analysis, patients with missing data at subsequent timepoints were excluded at both timepoints. Further exclusion criteria for patient data were (a) missing clinical data at baseline (n = 39), and (b) missing data or invalid dates of completion of one of the HRQoL measures (n = 71).

To investigate potential selection bias we compared the selected data with original patient data at baseline and subsequent time points. Chi-squared tests were performed to check if data selection was biased in terms of sex, randomization group, and clinical subgroups (ECOG Performance Status (PS), steroid intake, extent of surgery). T-tests were performed to check bias of selected data in terms of age, time of progression-free survival and overall survival in days from randomization. Significant results (p ≤ 0.05) were considered signals for selection bias. 10% of all tested patient characteristic comparisons are expected to differ at significance level, which is here used as threshold for signaling bias [26].

## HRQOL measures

### EORTC QLU-C10D

The QLU-C10D utility score is calculated from 13 items of the QLQ-C30. Each item (e. g. “Have you had pain?”) has four response levels (1 = “Not at all” to 4 = “very much”). The 13 items form ten dimensions: Four covering aspects of functioning (Physical Functioning (PF), Role Functioning (RF), Social Functioning (SF), and Emotional Functioning ((EF)), and six covering symptoms (Pain (PA), Fatigue (FA), Sleep (SL), Appetite (AP), Nausea (NA), and Bowel Problems (BO)) commonly experienced by cancer patients.

### EQ-5D-3L

The EQ-5D-3L comprises of five dimensions: mobility (MO), self-care (SC), usual activities (UA), pain/discomfort (PD), and anxiety/depression (AD). Each dimension is presented with three response levels of severity (e.g. “I have no pain or discomfort” (Level 1), “I have moderate pain or discomfort” (Level 2), “I have extreme pain or discomfort” (Level 3)).

Index scales were calculated according to algorithms published for scoring the AUS [27, 28]) CAN [29, 30], UK [31, 32], and USA [33, 34] country-specific value sets of each measure.

### Known-groups

For cross sectional sensitivity analysis ECOG PS groups (0 vs. > 0) was available at baseline. It was previously shown to be associated with HRQoL deterioration in glioblastoma patients ([35]). Furthermore, the QLQ-C30 Global Health Status scale (GHS) was considered to reflect HRQoL groups in glioblastoma patients at baseline, with scores ≤ 50 vs. > 50 threshold, the “low mean” in cancer populations ([36]). The GHS is not part of the QLU-C10D items. The GHS is frequently reported as HRQoL score for patients with glioblastoma ([37]), and most importantly, is a common HRQoL outcome in cancer clinical trials (e. g. [38–42]). With regard to responsiveness we investigated changes over time in HRQoL of the total sample (overall change), clinical response groups (progressive vs. non-progressive) and changes measured with the GHS. Minimal important change over time of ± 6 GHS scores ([43]) was used to define GHS change groups (deterioration and improvement groups) vs. GHS stable group (< 6 scores change). Treatment arm was not used as a known-group factor, because no significant treatment effect was reported for HRQoL outcomes [25].

### Statistical analyses

Descriptive statistics are presented as means, standard deviations (SD), and frequencies.

## Validity

Floor and ceilings effects were calculated for index scales of the QLU-C10D and the EQ-5D-3L and for each of their domains. Frequencies of more than 15% at floor or ceiling levels were considered relevant, i.e. potentially limiting validity [44].

### Construct validity

Construct validity was analyzed by assessing correlations and agreement between the cancer-specific QLU-C10D and the generic EQ-5D-3L. The EQ-5D-3L served as a comparator for construct validity, because it is a well validated measure and recommended by health-care authorities [15, 17, 21]. Both measures were developed to measure HRQoL utility, hence a certain level of agreement was expected, but due to the different designs and scopes, differences should also be detectable.

Overall we expected high agreement between both measures, because of the similar construct. At the domain level we expected both high and low correlations (convergent and divergent validity) between QLU-C10D and EQ-5D-3L, because the QLU-C10D comprises of rather generic and cancer-specific domains. We expected moderate to strong correlations between conceptually similar domains (PF and MO/UA, RF and UA, SF and UA, PA and PD, EF and AD, FA and UA), which would indicate that these domains address related HRQoL concepts. However, rather disease-specific domains of the QLU-C10D were not expected to relate with EQ-5D-3L domains to that extent. Hence low correlations between these QLU-C10D domains (SL, AP, NA, BP) and the EQ-5D-3L domains were expected, indicating that QLU-C10D also shows divergent validity from EQ-5D-3L because of the disease-specific domains [45]. Pearson correlation coefficients (r) between domains of the QLU-C10D and the EQ-5D-3L and intra-class correlations (ICC) for both index scales were calculated. Standard convention was used for interpreting r (< 0.50 signal weak, < 0.70 moderate and ≥ 0.70 strong correlations) [46]. ICC was computed based on pairs of observations of EQ-5D-3L and QLU-C10D raw scores and in country-wise index scales. ICC values for absolute agreement ≥ 0.75 indicate excellent agreement [47].

Bland–Altman plots were generated to analyze agreement between the QLU-C10D and the EQ-5D-3L. The visual presentation of differences between the measures and best estimate of the true value (mean of both measures) allowed detailed identification of (dis-)agreements of both measures. The difference between both measures is plotted on the y-axis and the average on the x-axis. Proportional bias was analyzed with linear regressions, using means to predict the difference of both measures. Significant F-values indicate the presence of proportional bias. Lacking country-specific minimal important difference (MID) for glioblastoma patients for the other value sets, we used MID reported for the EQ-5D-3L UK value set in glioma patients as pre-defined acceptable levels of agreement (LOA) of ± 0.15 [48].

### Sensitivity

The ability to discriminate between patient groups was assessed by calculating independent t-tests across clinically known-groups. These were the ECOG PS (0 vs. > 0), and the GHS (≤ 50 vs. > 50) at baseline, and difference in change between clinical response groups (progressive vs non-progressive) at subsequent timepoints. A one-way analysis of variance (ANOVA) was calculated for differences in change between the three GHS change groups.

### Responsiveness

**Fig. 1:**
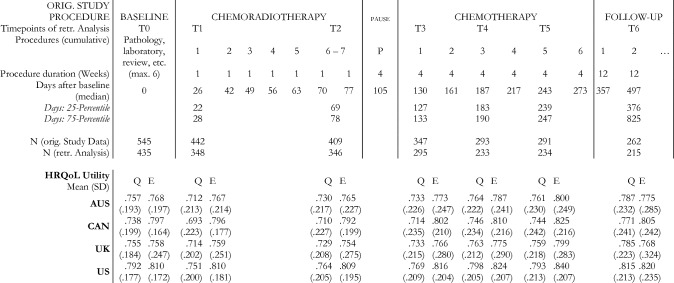
Original Study Procedures with assessment timepoints for HRQoL. Abbreviations: SD = Standard Deviation; HRQoL = Health-related Quality of Life; retr. = retrospective; Q = QLU-C10D; E = EQ-5D-3L; Country value set: AUS = Australia, CAN = Canada, UK = United Kingdom

Responsiveness was assessed for overall change, and within clinical response and GHS subgroups (baseline to Tx) using paired t-tests. Responsiveness for changes within clinical response subgroups were assessed between baseline and end of treatment at T5. Responsiveness for changes within GHS change subgroups was analyzed for the periods between baseline and two time-points during chemoradiotherapy (T1 & T2 = first and last timepoint of chemoradiotherapy), three timepoints during chemotherapy (T3-T5), and at follow-up assessment (T6) (see Fig. 1).

## Effect size measures

Effect size of between-group differences, Cohen’s d, was calculated by dividing the mean difference with the pooled SD. Magnitude of effect size was interpreted according to Cohen’s guidelines [49], i.e. ≥ 0.5 were considered medium and ≥ 0.8 large.

Magnitude of change over time within the total sample was estimated with Standardized Response Mean (SRM), which standardizes mean change with the SD of change. The Responsiveness Index (RI) was calculated as a quotient of SD of stable or non-progressive groups as denominator, and within-group change (progressive, decrease, increase) as numerator.

To compare value sets of both measures directly in terms of statistical efficiency, we calculated Relative Efficiency (RE) as a ratio of positive t-values or F-values with the QLU-C10D in the numerator and the EQ-5D-3L in the denominator. Results > 1 indicate a higher efficiency of the QLU-C10D, and results < 1 indicate a higher efficiency of the EQ-5D-3L. RE was only calculated if at least one of the measures found a statistically significant effect. Further, differences in effect sizes were calculated, under the same premise as RE calculations. Difference of RI (DRI) was calculated to compare HRQoL measures in terms of within-groups responsiveness. DRI was calculated by subtracting positive RI-values of the EQ-5D-3L from positive RI-values of the QLU-C10D [50]. Likewise, the difference of Cohen’s d and SRM was calculated. Results > 0 indicate a higher responsiveness of the QLU-C10D, and < 0 favor the EQ-5D-3L. A maximal probability of error 1% (i.e. a p-value ≤ 0.01) was used to keep alpha error accumulation at a minimum [51].

## Results

### Descriptive statistics and *bias* analyses

The QLQ-C30 and EQ-5D-3L were completed by 435 of 545 patients at baseline, and 215 of 262 at follow-up (= T6). An overview of sociodemographic and clinical characteristics is presented in Table 1. Mean utilities of the QLU-C10D value sets at baseline were between 0.738 and 0.792. Between 15 and 22% of the original study could not be included due to missing data at different time points. With the exception of steroid intake (baseline, T2, T4, T5) and age (T4, T5, T6), no significant differences between selected data and the original sample were found. These exceptions account for less than 10% of expected differences, and therefore are considered negligible. An overview of bias analysis is included in Appendix E.

**Table 1:** Sample Characteristics at baseline (N = 435)

**Age** (mean)/(SD); (median)	56,2/11	57.9
**Sex:** Male: (n); (%)	236	54.3
Female: (n); (%)﻿	199	45.7
**Steroid at baseline:** No: (n); (%)﻿	268	61.6
Yes: (n); (%)﻿	167	38.4
**Treatment:** Chemoradiotherapy: (n); (%)﻿	218	50.1
Added Cilengitide: (n); (%)﻿	217	49.9
**ECOG** Performance status 0: (n); (%)﻿	252	57.9
Performance status > 0: (n); (%)﻿	183	42.1
**Extent of surgery:** Biopsy: (n); (%)﻿	14	3.2
Partial resection: (n); (%)﻿	211	48.5
Complete resection: (n); (%)﻿	208	47.8
**Overall survival:** days (median); (SD)	710	318.6
**Progression free survival:** days (median); (SD)	255	310.3

### Floor & ceiling effects

Ceiling effect (proportion of patients reporting full health) on the QLU-C10D index scale was 4.14%. The EQ-5D-3L index scale showed higher ceiling effects (27.13%).

The largest ceiling effect of the QLU-C10D domains was NA, at 88.05%, and the smallest proportion was SF, at 27.59%. The EQ-5D-3L domains MO (73.56%) and UA (51.49%) exhibit higher ceiling effects than the conceptually similar QLU-C10D domains (PF: 46.90%, RF: 36.63%, and SF: 27.58%). Floor and ceiling effects of index scores and response levels per domain are presented in Tables 2 and 3.

**Table 2:** Floor & Ceiling Effects Index Scores at Baseline (Raw & Country value set)

BASELINE (N = 435)	Floor	n / %	Ceiling	n / %
**QLU-C10D** (raw)	40	0 / 0	10	18 / 4.14
AUS	− 0.10	0 / 0	1.00	29 / 6.67
CAN	− 0.15	0 / 0	1.00	18 / 4.14
UK	− 0.08	0 / 0	1.00	18 / 4.14
USA	0.03	0 / 0	1.00	18 / 4.14
**EQ-5D-3L** (raw)	15	0 / 0	5	118 / 27.13
AUS	− 0.22	0 / 0	1.00	118 / 27.13
CAN	− 0.31	0 / 0	1.00	118 / 27.13
UK	− 0.59	0 / 0	1.00	118 / 27.13
USA	− 0.11	0 / 0	1.00	118 / 27.13

Floor health state for QLU-C10D = 44444 44444, EQ-5D-3L = 33333; Ceiling health state for QLU-C10D = 11111 11111, EQ-5D-3L = 11111; Lowest health states observed for QLU-C10D = 34444 44444 (utility value AUS = 0.004; CAN = − 0.053; UK = 0.016; USA = 0.151), EQ-5D-3L = 33232 (utility value AUS = − 0.086; CAN = − 0.088; UK = − 0.371; USA = − 0.012)

Abbreviations Country value set: AUS = Australia, CAN = Canada, UK = United Kingdom, USA = United States of America

**Table 3:** Floor & Ceiling Effects Single Domains EQ-5D-3L & QLU-C10D raw scoring (N = 435)

EQ-5D-3L	MO — Mobility		SC — Self-Care		UA — Usual Activities		PD — Pain / Discomfort		AD — Anxiety / Depression	
level	n	%	n	%	n	%	n	%	n	%
1	320	73.56	368	84.60	224	51.49	295	67.82	213	48.96
2	112	25.75	60	13.79	168	38.62	135	31.03	202	46.44
3	3	0.69	7	1.61	43	9.89	5	1.15	20	4.60

QLU-C10 Dimensions	PF		RF		SF		EF		PA		FA		SL		AP		NA		BP	
Level	n	%	n	%	n	%	n	%	N	%	n	%	n	%	n	%	n	%	n	%
1	204	46.90	168	36.63	120	27.59	202	46.43	283	65.05	119	27.36	212	48.74	349	80.23	383	88.05	291	66.90
2	132	30.34	146	33.56	159	36.55	165	37.93	121	27.82	223	51.26	143	32.87	56	12.87	44	10.11	107	24.60
3	69	15.86	76	17.47	98	22.53	43	9.89	25	5.75	68	15.63	58	13.33	23	5.29	4	0.92	26	5.98
4	30	6.90	45	10.34	58	13.33	25	5.75	6	1.38	25	5.75	22	5.06	7	1.61	4	0.92	11	2.52

Abbreviations: PF = Physical Functioning; RF = Role Functioning; SF = Social Functioning; EF = Emotional Functioning; PA = Pain; FA = Appetite; NA = Nausea; BP = Bowel Problems

### Construct validity

The four country value set of each HRQoL measure produced similar correlation coefficients, presented in Table 4. ICC coefficients of index scales of all country value set va indicate strong agreement, ranging between 0.718 and 0.750. Mostly moderate positive correlations were found between conceptually similar domains, ranging between 0.329 and 0.632, and weak correlations (r < 0.291) between the cancer-specific QLU-C10D and the generic EQ-5D-3L domains. Collectively the correlations between QLU-C10D and EQ-5D-3L domains indicate that correlation coefficients increase with content-wise proximity of the concepts.

**Table 4:** Correlations between QLU-C10D and EQ-5D-3L Index Scales and Domains at baseline (N = 435)

		Domains (Pearson’s r)									
		1	2	3	4	5	6	7	8	9	10
*QLU-C10D*	Index Scales (ICC)	PF	RF	SFg	EF	PA	FA	SL	AP	NA	BP
*EQ-5D-3L*		MO, UA	UA	UA	AD	PD	UA	All	All	All	All
Expected Correlation	Strong ≥ 0.7	Strong, moderate > 0.5	Moderate ≥ 0.5	Moderate ≥ 0.5	Strong ≥ 0.7	Strong ≥ 0.7	Moderate < 0.7	Weak < 0.5	Weak < 0.5	Weak < 0.5	Weak < 0.5
AUS	.749	.496*	.615	.467	.632	.555	.329	< .171	< .146	< .291	< .269
CAN	.734	.483*	.601	.466	.614	.565	.396	< .171	< .172	< .255	< .270
UK	.718	.497*	.615	.496	.601	.601	.338	< .228	< .157	< .278	< .248
USA	.750	.495*	.598	.479	.570	.594	.411	< .219	< .165	< .283	< .278

All correlations are significant with p-values < .01

*Abbreviations: *ICC = Intra-Class Correlations;  AD = Anxiety, Depression; AP = Appetite; BP = Bowel Problems; EF = Emotional Functioning; FA = Fatigue; MO = Mobility; NA = Nausea; PA = Pain; PD = Pain, Discomfort; PF = Physical Functioning; RF = Role Functioning; SF = Social Functioning; SL = Sleep; UA = Usual Activities; Country value sets: AUS = Australia, CAN = Canada; UK = United Kingdom; USA = United States of America

*Smallest correlation with the two EQ-5D-3L domains

**Fig. 2:**
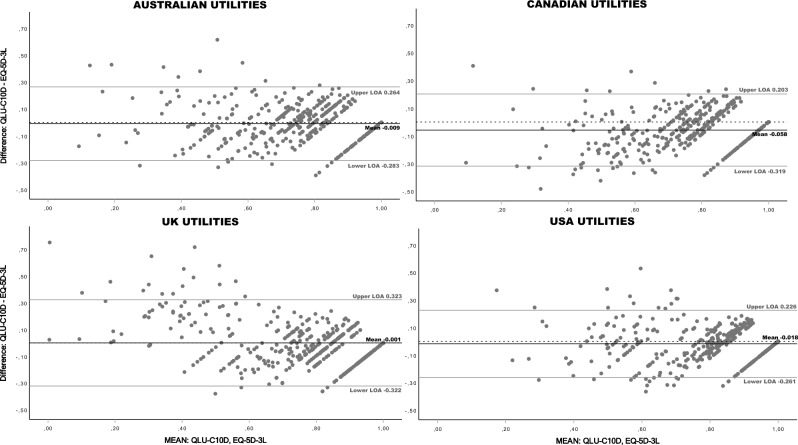
Bland–Altman Plots comparing QLU-C10D and EQ-5D-3L Country value sets at baseline. Dotted horizontal line = 0 at y-axis; LOA = Level of Agreement (= mean ± 1.96 * SD); *Proportional bias:* Results of Linear regressions, predicting differences with means (df = 1, 433): AUSTRALIAN Utilities, r = .09, R^2^ = .008, F = 3.54, p = .061; CANADIAN Utilities, r = .229, R^2^ = .052, F = 23.96, p < .001; UK Utilities, r = .444, R^2^ = .197, F = 106.10, p < .001; USA Utilities, r = .001, R^2^ = .000, F = 0.0, p = .992; Regression lines are not displayed

Bland–Altman plots of index scores showed a negative bias, indicating that the QLU-C10D scores tend to be lower than the EQ-5D-3L scores with all value sets (Fig. 2). Mean bias ranged from -0.058 (CAN) to -0.001 (UK). Levels of agreement (LOA) exceeded the pre-defined level of ± 0.15 for all value sets. Furthermore, Bland–Altman plots indicate that the extent of agreement differs across the measurement range in all value sets, i.e. dispersion increases towards the lower end of the scale. Proportional bias was detected with two value set pairs.

### Sensitivity

**Fig. 3:**
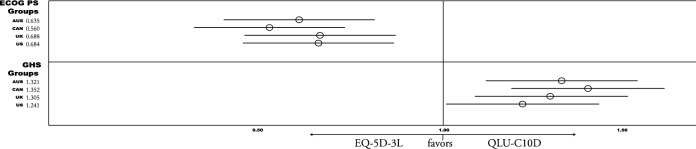
Relative Efficiency (RE) of Country value sets for detecting known-groups at baseline. Abbreviations *Country value sets:* AUS = Australia, CAN = Canada, UK =United Kingdom, US =United States of America; Known Groups: ECOG Performance status (PS) 0 vs. > 0; QLQ-C30 Global Health Status Score (GHS)  ≤ 50 vs. > 50; RE results > 1 favor QLU-C10D, < 1 favor EQ-5D-3L. Descriptive & detailed results see Appendix A

Results of sensitivity analysis show that both measures detected significant medium to large differences when categorizing patients by ECOG PS (0 vs. > 0), and the GHS (≤ 50 vs. > 50), with all value sets. The EQ-5D-3L showed higher sensitivity for ECOG groups (Cohen’s d > 0.75). The QLU-C10D detected larger differences between GHS groups (Cohen’s d > 1.14). These results are reflected in RE of the four value set comparisons, which favor the EQ-5D-3L for ECOG groups (RE < 0.69), and the QLU-C10D for GHS groups (RE > 1.24) (see Fig. 3 or detailed statistics in Appendix A).

### Responsiveness

**Fig. 4:**
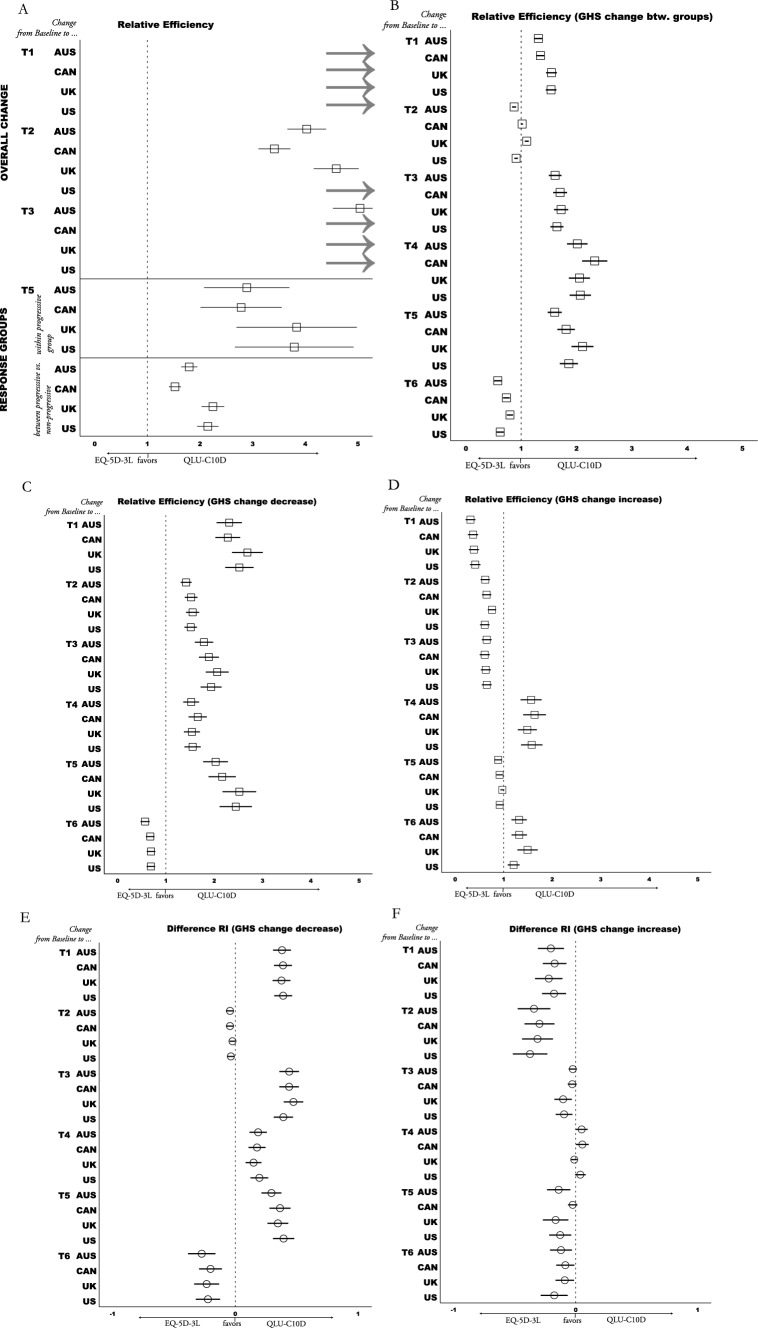
Responsiveness Analysis QLU-C10D vs. EQ-5D-3L. Grey arrows signify values outside the scope of x-axis; *Abbreviations:* Country value sets*:* AUS = Australia, CAN = Canada, UK = United Kingdom, US = United States of America; RE = Relative Efficiency; GHS = Global Health Scale (QLQ-C30); **A** RE = quotient of t-values of paired t-tests (Tx—Baseline); **B** RE = quotient of f-values of one-way ANOVA, comparing QLQ-C30 GHS groups (> 6 points decrease vs. ≤ 6 points change vs. > 6 points increase); **C** & **D** RE = quotient of t-values calculated with paired t-tests (T_x_—Baseline); **E** & **F** Responsiveness Index (RI) standardizes mean Decrease or Increase of GHS groups with the standard deviation of the stable group; Difference RI = RI QLU-C10D minus RI EQ-5D-3L; Details of calculations and results of A are presented in Appendix B, B-F in Appendix C

Significant overall change within the total sample (paired t-tests of all patients) from baseline to T1 and to T3 (first chemoradiation and first chemotherapy) was detected only with the QLU-C10D. No significant changes with either measure were detected from baseline to later assessments. Twelve country value set pair comparisons (= 3 periods × 4 value set pairs) of both measures show a) larger SRM effects are detected with the QLU-C10D than with EQ-5D-3L value sets (SRM − 0.26 to − 0.17 vs. − 0.04 to 0.04), and b) RE results (> 3.42) also favored the QLU-C10D (see Fig. 4A; detailed results are presented in Appendix B).

All QLU-C10D value sets detected significant changes within progressive patients (RI < − 0.56) as well as differences in change between progressive and non-progressive patients (Cohen’s d > − 0.55). These effects were not detected by the EQ-5D-3L. All eight value set comparisons (4 within progressive subgroup, 4 between response groups) favored the QLU-C10D (DRIs ≥ 0.28, REs ≥ 1.53; see detailed results in Appendix D).

Significant results were observed within both decreasing and increasing GHS change groups, with the exception of the increase group at T1 when measured with the QLU-C10D. These findings consistently aligned with the expected direction when compared to the baseline, across all time points and using both measures including no significant changes in the stable group (see Appendix C for details). Twenty-four comparisons (= 6 periods × 4 value set pairs) for each group were performed. In total, 20 of 24 RE calculations (> 1.42) of the decreasing group favored the QLU-C10D (see Fig. 4C). Also, 16 of 24 DRI calculations of decreasing GHS change groups favored the QLU-C10D (see Fig. 4E). For calculations of increasing GHS change groups, 16 of 24 RE and 20 of 24 DRI results favored the EQ-5D-3L (seeFig. 4D and F). Difference between mean change of GHS change groups (decreasing, increasing, stable) was significant at all time points with both HRQoL measures (see Appendix C). Twenty-four comparisons (= 6 periods × 4 value set pairs) show that 18 RE calculations (> 1.02) favor QLU-C10D (see Fig. 4B).

In summary, with regard to responsiveness the QLU-C10D showed favorable characteristics and outperformed the EQ-5D-3L in most comparisons of the four value sets (71.7%). Especially in the detection of differences between GHS change subgroups (75.0%), clinical response groups (100.0%), of changes within progressive group (100.0%) and deteriorating GHS subgroup (83.3%) favorable results have been shown for the QLU-C10D.

## Discussion

The aim of this analysis was to assess the validity of the QLU-C10D in patients with glioblastoma with four value sets – AUS, CAN, UK, and USA. To our knowledge, this is the first validation of the QLU-C10D in this patient population [52, 53].

Overall, results of our analysis indicate that the QLU-C10D can be considered a clinically valid utility measure in newly diagnosed glioblastoma patients. A range of validity indicators point to this conclusion.

Firstly, we investigated construct validity on multiple levels. Floor or ceiling effects are considered signals for limited validity. The QLU-C10D index scale showed negligible ceiling effects. The highest ceiling effects in QLU-C10D single domains showed AP, NA, BP and PA, which are not common symptoms of patients with glioblastoma in early stages of disease [2, 3, 5]. Conceptually similar domains with EQ-5D-3L, Mobility and Physical Functioning, showed divergence in floor and ceiling levels. Similar results of this domain pair were previously reported [54, 55]. This indicates that the underlying concepts of the conceptually similar domains are not identical.

Indeed, this assumption was supported by the results of correlation analyses. This analysis overall showed expected results: QLU-C10D and EQ-5D-3L index scales showed strong agreement, conceptually similar domains indicated convergent validity, while conceptually distant domains (EQ-5D-3L domains and QLU-C10D cancer-specific symptom domains) showed divergent validity. Yet, the Emotional Functioning scale of the QLU-C10D and the anxiety/depression domain of the EQ-5D-3L, as well as the pain scales of both instruments, showed lower correlations than expected. Unlike the QLU-C10D domains, AD and PD are EQ-5D-3L composite domains, where previous research has shown that splitting these domains up in separate items, e. g. one item for anxiety and one separate item for depression, reduced the number of “no problem” reports on this domain [56]. Another source of divergence are different scale levels, which contribute to limited correlations of conceptually similar domains. For example the most severe level at Physical Function is labelled “severe problems” at the QLU-C10D, and “confined to bed” at the EQ-5D-3L Mobility domain. Given that an updated version of the EQ-5D with five response options instead of three is being used increasingly, comparisons with this novel version might provide more insight into this discrepancy [19].

Secondly, we assessed QLU-C10D performance in detecting known groups and changes over time as core psychometric properties of a HRQoL measure. The QLU-C10D performed well in the majority of tests. It was able to detect ECOGS PS groups, HRQoL groups measured with the QLQ-C30 GHS, changes within progressive patients, and correctly measured direction of GHS change (e. g. deteriorated, improved). The latter is of special interest, as the QLQ-C30 GHS is frequently used as an HRQoL outcome in cancer clinical trials (e. g. [37–41]), and it might be considered a minimum requirement of the QLU-C10D to detect HRQoL changes measured with that scale. Our consistent finding on the stable HRQoL group (i.e. no change measured by GHS and no change detected by the QLU-C10D) is in contrast with Shaw et al. [57], who reported significant changes in QLU-C10D scores in stable HRQoL groups in melanoma and head & neck cancer patients.

In direct comparison with the generic EQ-5D-3L our analyses showed that the QLU-C10D was more sensitive in detecting differences in GHS groups while the EQ-5D-3L showed higher sensitivity for ECOG groups. The latter is in contrast to previous research, reporting higher sensitivity of the QLU-C10D than the EQ-5D-3L for ECOG PS in myelodysplastic syndrome patients [45], mixed cancer samples [57, 58], and lung cancer patients [59], all using the same cutoffs as the here presented analysis. This discrepancy with the previous research was surprising. A potential reason could be that in this patient population the EQ-5D-3L self-care domain covers aspects of categorization which is reflected in sensitivity for ECOG PS. With regard to responsiveness the QLU-C10D outperformed the EQ-5D-3L in most comparisons (71.7%). Previous research likewise has indicated higher responsiveness of QLU-C10D than EQ-5D-3L [57–59].

Finally, on the topic of country differences without a potential language bias, our secondary aim, we found that the four QLU-C10D value sets largely perform the same way, meaning that although utility weights of single domains vary, effect sizes and directions calculated with these value sets are mostly highly similar.

### Limitations and critical appraisal

The main limitation of our analyses is their retrospective nature. Group comparisons therefore were guided by the availability of clinical information, while other potentially relevant information could not be retrieved. It was therefore also only possible to use the three level version of the EQ-5D as comparator, which still is the preferred generic utility instrument in many jurisdictions [21, 60]. To learn more about the differences between cancer-specific and generic utilities future investigations of the QLU-C10D require not only comparison with the novel EQ-5D version but also with other, generic utility instruments.

We also had to exclude patients from analyses due to missing HRQoL data. Although we performed a bias analysis with regard to available sociodemographic and clinical information, we cannot fully rule out that more severely HRQoL-impaired patients dropped out of assessment. This risk might be indicated by the bias analysis, where at some timepoints we found a significantly higher proportion of patients with steroid intake and higher mean age in the original sample than in the data for this analysis. However, we found no significant HRQoL differences with factors age or steroid intake. Considering that significant differences in sample composition were found only in 7 of 77 (11 factors × 7 assessments) tests, we suggest that potential bias effects on this analysis are negligible.

Finally, lacking a better estimate we used the EQ-5D-3L MID for glioma patients with the UK value set as a measure of relevant difference in Bland–Altman plots (48). This MID might not necessarily be transferrable to the value sets of other countries as used here.

## Conclusion

In conclusion, our results suggest that the QLU-C10D—as a preference-based scoring algorithm for data collected with the QLQ-C30—is a valid measure for use in newly diagnosed glioblastoma patients. Hence, the QLQ-C30 can provide both, cancer-specific HRQoL health profiles and a utility score (QLU-C10D), and serve multiple purposes in cancer clinical trials and health economic studies in glioblastoma. The tendency of the QLU-C10D to be more sensitive to health changes, especially when it comes to detecting deteriorating HRQoL, translates into higher statistical power and consequently smaller required sample sizes.

## Supplementary Information

Below is the link to the electronic supplementary material.

Supplementary file1 (DOCX 90 KB)

## Data Availability

The data used for this analyses is owned by Merck and the EORTC. It cannot be shared by the authors of this publication. An official request though can be made with the EORTC. See EORTC Policy on Data Sharing: https://www.eortc.org/app/uploads/2023/06/L-01-POL-01.pdf
