# Supplementary material for: Cancer-specific utility: clinical validation of the EORTC QLU-C10D in patients with glioblastoma
Source: Eur J Health Econ. 2024 Nov 20;26(5):721–33. doi: 10.1007/s10198-024-01729-4 (PMC12204889; doi:10.1007/s10198-024-01729-4)
Supplement: Supplementary file 1 — Supplementary file1 (DOCX 90 KB) [file 10198_2024_1729_MOESM1_ESM.docx]

Appendix A: Sensitivity

| **Appendix A:** Sensitivity for ECOG Performance Status (PS) and Global Health Status Scale (QLQ-C30) | | | | | | | | | | |
| --- | --- | --- | --- | --- | --- | --- | --- | --- | --- | --- |
| Baseline  (N = 435) | Mean | SD | Mean | SD | Mean Diff.^†^ | SDp | t | p | Cohen’s d [95% CI] | RE* [95% CI] |
| ECOG | PS 0  N = 252 |  | PS > 0  N = 183 |  |  |  |  |  |  |  |
| AUS QLU-C10D | .801 | .185 | .691 | .181 | -.110 | .183 | 6.166 | .000 | -.599 [-.793; -.404] | .635  [.444; .826] |
| AUS EQ5D | .824 | .176 | .681 | .208 | -.143 | .190 | 7.739 | .000 | -.752 [-.948; -.555] |  |
| CAN QLU-C10D | .781 | .191 | .674 | .186 | -.107 | .189 | 5.854 | .000 | -.569 [-.763; -.375] | .560  [.369; .751] |
| CAN EQ5D | .844 | .148 | .725 | .168 | -.119 | .157 | 7.825 | .000 | -.760 [-.957; -.563] |  |
| UK QLU-C10D | .797 | .176 | .692 | .173 | -.104 | .175 | 6.144 | .000 | -.597 [-.791; -.402] | .688  [.496; .879] |
| UK EQ5D | .828 | .206 | .651 | .271 | -.177 | .235 | 7.409 | .000 | -.751 [-.948; -.554] |  |
| US QLU-C10D | .832 | .164 | .730 | .174 | -.103 | .168 | 6.210 | .000 | -.609 [-.803; -.414] | .684  [.492; .875] |
| US EQ5D | .859 | .146 | .735 | .187 | -.125 | .165 | 7.510 | .000 | -.758 [-.955; -.561] |  |
| GHS | ≤ 50  N = 136 |  | > 50  N = 299 |  |  |  |  |  |  |  |
| AUS QLU-C10D | .619 | .184 | .817 | .160 | .197 | .168 | 10.798 | .000 | 1.176 [.958; 1.393] | 1.321  [1.100; 1.541] |
| AUS EQ5D | .646 | .217 | .818 | .171 | .172 | .186 | 8.177 | .000 | .923 [.712; 1.135] |  |
| CAN QLU-C10D | .596 | .190 | .799 | .163 | .203 | .172 | 10.805 | .000 | 1.181 [.964; 1.398] | 1.352  [1.130; 1.573] |
| CAN EQ5D | .700 | .174 | .837 | .146 | .137 | .155 | 7.994 | .000 | .883 [.672; 1.094] |  |
| UK QLU-C10D | .625 | .174 | .811 | .154 | .186 | .161 | 10.734 | .000 | 1.160 [.943; 1.377] | 1.305  [1.084; 1.525] |
| UK EQ5D | .600 | .286 | .823 | .197 | .223 | .229 | 8.226 | .000 | .973 [.760; 1.186] |  |
| US QLU-C10D | .668 | .175 | .845 | .146 | .177 | .156 | 10.282 | .000 | 1.136 [.920; 1.353] | 1.241  [1.022; 1.460] |
| US EQ5D | .701 | .194 | .855 | .143 | .154 | .161 | 8.283 | .000 | .959 [.746; 1.171] |  |
|  |  |  |  |  |  |  |  |  |  |  |
| * RE is quotient of t-values of independent t-tests (= QLU-C10D_tariff_ / EQ-5D-5L_tariff_);  † Mean Difference = (mean PS > 0; GHS > 50) – (mean PS 0; GHS ≤ 50);  *Abbreviations:* GHS = Global Health Status Scale (QLQ-C30); t = t-value; p = p-value of t-tests; SD = Standard Deviation; RE = Relative Efficiency; CI = estimated 95% Confidence Interval. | | | | | | | | | | |

# Appendix B: Responsiveness Overall

| **Appendix B:** Responsiveness for change from Baseline to subsequent Timepoints (total sample) | | | | | | | | | | |
| --- | --- | --- | --- | --- | --- | --- | --- | --- | --- | --- |
|  | Baseline  Mean SD | | T_x_  Mean SD | | Mean Change* | SD | t | p | SRM [95% CI] | RE [95% CI] |
| T1  (N = 348) |  |  |  |  |  |  |  |  |  |  |
| AUS QLU-C10D | .757 | .193 | .712 | .213 | **-.045** | .172 | -4.824 | .000 | -.259 [-.408; -.109] | 30.532  [27.377; 33.686] |
| AUS EQ5D | .768 | .197 | .767 | .214 | -.002 | .187 | -.158 | .874 | -.008 [-.157; .140] |  |
| CAN QLU-C10D | .738 | .199 | .693 | .223 | **-.045** | .180 | -4.648 | .000 | -.249 [-.398; -.100] | 42.255  [37.868; 46.641] |
| CAN EQ5D | .797 | .164 | .796 | .177 | -.001 | .160 | -.110 | .912 | -.006 [-.154; .143] |  |
| UK QLU-C10D | .755 | .184 | .714 | .202 | **-.041** | .162 | -4.749 | .000 | -.255 [-.404; -.105] | 63.320  [56.720; 69.920] |
| UK EQ5D | .758 | .247 | .759 | .251 | .001 | .228 | .075 | .941 | .004 [-.145; .153] |  |
| US QLU-C10D | .792 | .177 | .751 | .200 | **-.041** | .163 | -4.638 | .000 | -.249 [-.398; -.099] | 90.941  [81.439; 100.443] |
| US EQ5D | .810 | .172 | .810 | .181 | .000 | .162 | -.051 | .959 | -.003 [-.151; .146] |  |
| T2  (N = 346) |  |  |  |  |  |  |  |  |  |  |
| AUS QLU-C10D | .762 | .189 | .730 | .217 | **-.032** | .183 | -3.230 | .001 | -.174 [-.323; -.024] | 4.027  [3.660; 4.395] |
| AUS EQ5D | .775 | .199 | .765 | .227 | -.009 | .213 | -.802 | .423 | -.043 [-.192; .106] |  |
| CAN QLU-C10D | .742 | .194 | .710 | .227 | **-.032** | .186 | -3.196 | .002 | -.172 [-.321; -.023] | 3.415  [3.112; 3.717] |
| CAN EQ5D | .802 | .166 | .792 | .199 | -.010 | .189 | -.936 | .350 | -.050 [-.199; .099] |  |
| UK QLU-C10D | .759 | .181 | .729 | .208 | **-.030** | .174 | -3.253 | .001 | -.175 [-.324; -.026] | 4.588  [4.161; 5.016] |
| UK EQ5D | .765 | .247 | .754 | .275 | -.010 | .268 | -.709 | .479 | -.038 [-.187; .111] |  |
| US QLU-C10D | .795 | .173 | .764 | .205 | **-.031** | .172 | -3.306 | .001 | -.178 [-.327; -.028] | 5.584  [5.051; 6.118] |
| US EQ5D | .815 | .174 | .809 | .195 | -.006 | .190 | -.592 | .554 | -.032 [-.181; .117] |  |
| T3  (N = 269) |  |  |  |  |  |  |  |  |  |  |
| AUS QLU-C10D | .766 | .177 | .733 | .226 | **-.033** | .194 | -2.969 | .003 | -.173 [-.335; -.011] | 5.041  [4.526; 5.556] |
| AUS EQ5D | .781 | .197 | .773 | .247 | -.008 | .242 | -.589 | .556 | -.034 [-.196; .127] |  |
| CAN QLU-C10D | .748 | .182 | .714 | .235 | **-.035** | .203 | -2.933 | .004 | -.171 [-.332; -.009] | 5.534  [4.962; 6.106] |
| CAN EQ5D | .808 | .165 | .802 | .210 | -.006 | .209 | -.530 | .597 | -.031 [-.192; .131] |  |
| UK QLU-C10D | .765 | .169 | .733 | .215 | **-.033** | .184 | -3.035 | .003 | -.177 [-.338; -.015] | 8.159  [7.287; 9.031] |
| UK EQ5D | .773 | .242 | .766 | .280 | -.006 | .285 | -.372 | .710 | -.022 [-.183; .140] |  |
| US QLU-C10D | .800 | .163 | .769 | .209 | **-.031** | .179 | -2.999 | .003 | -.175 [-.336; -.013] | 6.770  [6.057; 7.483] |
| US EQ5D | .821 | .170 | .816 | .204 | -.005 | .206 | -.443 | .658 | -.026 [-.187; .136] |  |
| T4  (N = 233) |  |  |  |  |  |  |  |  |  |  |
| AUS QLU-C10D | .782 | .168 | .764 | .222 | -.017 | .213 | -1.236 | .218 | -.081 [-.263; .101] |  |
| AUS EQ5D | .791 | .188 | .787 | .241 | -.005 | .236 | -.307 | .759 | -.020 [-.202; .161] |  |
| CAN QLU-C10D | .765 | .174 | .746 | .234 | -.019 | .225 | -1.292 | .198 | -.085 [-.266; .097] |  |
| CAN EQ5D | .815 | .160 | .810 | .216 | -.005 | .215 | -.363 | .717 | -.024 [-.205; .158] |  |
| UK QLU-C10D | .781 | .158 | .763 | .212 | -.018 | .204 | -1.339 | .182 | -.084 [-.266; .097] |  |
| UK EQ5D | .785 | .229 | .775 | .290 | -.010 | .279 | -.554 | .580 | -.036 [-.218; .145] |  |
| US QLU-C10D | .816 | .151 | .798 | .205 | -.018 | .199 | -1.404 | .162 | -.092 [-.274; .090] |  |
| US EQ5D | .830 | .163 | .824 | .207 | -.005 | .200 | -.402 | .688 | -.026 [-.208; .155] |  |
| T5  (N = 234) |  |  |  |  |  |  |  |  |  |  |
| AUS QLU-C10D | .784 | .166 | .761 | .230 | -.023 | .210 | -1.709 | .089 | -.112 [-.293; .070] |  |
| AUS EQ5D | .794 | .190 | .800 | .249 | .007 | .258 | .396 | .692 | .026 [-.155; .207] |  |
| CAN QLU-C10D | .767 | .172 | .744 | .242 | -.023 | .220 | -1.623 | .106 | -.106 [-.287; .075] |  |
| CAN EQ5D | .817 | .160 | .825 | .216 | .008 | .225 | .524 | .601 | .034 [-.147; .215] |  |
| UK QLU-C10D | .783 | .156 | .759 | .218 | -.024 | .198 | -1.873 | .062 | -.122 [-.304; .059] |  |
| UK EQ5D | .790 | .230 | .799 | .283 | .009 | .302 | .469 | .639 | .031 [-.151; .212] |  |
| US QLU-C10D | .817 | .150 | .793 | .213 | -.025 | .199 | -1.896 | .059 | -.124 [-.305; .057] |  |
| US EQ5D | .832 | .164 | .840 | .207 | .008 | .219 | .590 | .556 | .039 [-.143; .220] |  |
| T6  (N = 215) |  |  |  |  |  |  |  |  |  |  |
| AUS QLU-C10D | .780 | .172 | .787 | .232 | .007 | .215 | .498 | .619 | .034 [-.155; .223] |  |
| AUS EQ5D | .788 | .193 | .775 | .285 | -.013 | .283 | -.688 | .492 | -.047 [-.236; .142] |  |
| CAN QLU-C10D | .763 | .177 | .771 | .241 | .008 | .223 | .553 | .581 | .038 [-.151; .227] |  |
| CAN EQ5D | .812 | .163 | .805 | .242 | -.007 | .245 | -.403 | .687 | -.027 [-.217; .162] |  |
| UK QLU-C10D | .778 | .162 | .785 | .223 | .007 | .206 | -.526 | .599 | .035 [-.154; .224] |  |
| UK EQ5D | .781 | .238 | .768 | .324 | -.013 | .330 | .507 | .613 | -.039 [-.228; .150] |  |
| US QLU-C10D | .814 | .155 | .815 | .213 | .001 | .199 | .105 | .916 | .007 [-.182; .196] |  |
| US EQ5D | .826 | .167 | .820 | .235 | -.006 | .238 | -.382 | .703 | -.026 [-.215; .163] |  |
|  |  |  |  |  |  |  |  |  |  |  |
| * Mean Change = Tx-score – Baseline-score; significant results (p ≤ .01) are emphasized in **bold**;  *Abbreviations:* T_x_ = Timepoint after baseline; SD = Standard Deviation; t = t-value of paired t-test; p = p-value of t-tests; SRM (standardized Response Mean) = mean change / SD change; RE (Relative Efficiency) = t-value_QLU-C10D_ / t-value_EQ-5D_; CI = estimated 95% Confidence Interval. | | | | | | | | | | |

Appendix C: Responsiveness (decreasing, increasing, stable) QLQ-C30 global health score

| Appendix C: Responsiveness QLQ-C30 Global Health Status Scale (stable, decreasing, increasing) | | | | | | | | | | | | | | | | |
| --- | --- | --- | --- | --- | --- | --- | --- | --- | --- | --- | --- | --- | --- | --- | --- | --- |
| *Tariffs & Timepoints* | **Stable** (≤ 6 score change) | | | | **Decrease** (> 6 score points) | | | | | **Increase** (> 6 score points) | | | | | **Betw. Groups** | |
|  |  |  |  |  |  | | RI |  | DRI |  | | RI |  | DRI | F |  |
|  | Mean | SD | t | p | mean | SD | t* | p | RE* | mean | SD | t* | p | RE* | p | RE** |
| **Australia** |  |  |  |  |  |  |  |  |  |  |  |  |  |  |  |  |
| QLU-C10D baseline | .806 | .172 |  |  | .752 | .189 |  |  |  | .710 | .212 |  |  |  |  | **N** |
| T1 (n = 91 / 172 / 83) | .779 | .185 |  |  | .668 | .220 | -.641 |  | .380 | .727 | .212 | .132 |  | -.204 | 10.630 | 346 |
| Change^†^ | -.027 | .130 | -1.971 | .052 | -.083 | .174 | -6.277 | .000 | 2.311 | .017 | .192 | .813 | .418 | .318 | .000 | 1.312 |
| T2 (n = 68 / 179 / 99) | .763 | .207 |  |  | .688 | .221 | -.509 |  | -.044 | .782 | .200 | .309 |  | -.342 | 20.035 | 346 |
| Change^†^ | -.013 | .166 | -.668 | .506 | -.085 | .176 | -6.437 | .000 | 1.420 | .051 | .174 | 2.934 | .004 | .621 | .000 | .873 |
| T3 (n = 57 / 150 / 86) | .826 | .200 |  |  | .657 | .236 | -.819 |  | .439 | .800 | .179 | .464 |  | -.024 | 28.829 | 293 |
| Change^†^ | .020 | .134 | 1.104 | .274 | -.110 | .197 | -6.818 | .000 | 1.789 | .062 | .167 | 3.462 | .001 | .652 | .000 | 1.614 |
| T4 (n = 41 / 117 / 73) | .853 | .162 |  |  | .679 | .250 | -.538 |  | .185 | .849 | .138 | .452 |  | .049 | 17.761 | 231 |
| Change^†^ | .043 | .182 | 1.517 | .137 | -.091 | .237 | -4.157 | .000 | 1.524 | .076 | .133 | 4.924 | .000 | 1.566 | .000 | 2.016 |
| T5 (n = 41 / 122 / 68) | .834 | .026 |  |  | .671 | .023 | -.659 |  | .294 | .889 | .013 | .661 |  | -.140 | 21.148 | 231 |
| Change^†^ | .010 | .141 | .465 | .645 | -.093 | .233 | -4.398 | .000 | 2.030 | .093 | .122 | 6.261 | .000 | .888 | .000 | 1.606 |
| T6 (n = 41 / 108 / 65) | .830 | .179 |  |  | .699 | .262 | -.268 |  | -.275 | .903 | .126 | .454 |  | -.121 | 9.861 | 214 |
| Change^†^ | .034 | .196 | 1.108 | .275 | -.052 | .251 | -2.174 | .032 | .571 | .089 | .112 | 6.397 | .000 | 1.319 | .000 | .578 |
| EQ-5D-3L baseline | .815 | .185 |  |  | .769 | .196 |  |  |  | .714 | .201 |  |  |  |  |  |
| T1 / RI | .836 | .178 |  |  | .728 | .214 | -.261 |  |  | .766 | .231 | .335 |  |  | 8.101 |  |
| Change^†^ | .021 | .156 | 1.275 | .206 | -.041 | .196 | -2.716 | .007 |  | .052 | .186 | 2.558 | .012 |  | .000 |  |
| T2 / RI | .826 | .161 |  |  | .709 | .253 | -.554 |  |  | .825 | .189 | .651 |  |  | 22.960 |  |
| Change^†^ | .024 | .138 | 1.407 | .164 | -.076 | .225 | -4.533 | .000 |  | .090 | .189 | 4.724 | .000 |  | .000 |  |
| T3 / RI | .851 | .225 |  |  | .701 | .271 | -.381 |  |  | .843 | .173 | .488 |  |  | 17.860 |  |
| Change^†^ | .013 | .212 | .451 | .654 | -.081 | .259 | -3.812 | .000 |  | .103 | .180 | 5.310 | .000 |  | .000 |  |
| T4 / RI | .885 | .147 |  |  | .714 | .278 | -.353 |  |  | .847 | .177 | .403 |  |  | 8.812 |  |
| Change^†^ | .029 | .184 | 1.010 | .319 | -.065 | .257 | -2.727 | .007 |  | .074 | .201 | 3.145 | .002 |  | .000 |  |
| T5 / RI | .887 | .028 |  |  | .716 | .026 | -.366 |  |  | .913 | .014 | .801 |  |  | 13.165 |  |
| Change^†^ | .028 | .160 | 1.116 | .271 | -.059 | .298 | -2.167 | .032 |  | .128 | .150 | 7.052 | .000 |  | .000 |  |
| T6 / RI | .854 | .187 |  |  | .656 | .326 | -.543 |  |  | .920 | .146 | .575 |  |  | 17.052 |  |
| Change^†^ | .039 | .210 | 1.196 | .239 | -.114 | .312 | -3.807 | .000 |  | .121 | .201 | 4.849 | .000 |  | .000 |  |
| **CanADA** |  |  |  |  |  |  |  |  |  |  |  |  |  |  |  |  |
| QLU-C10D baseline | .788 | .179 |  |  | .733 | .194 |  |  |  | .692 | .220 |  |  |  |  |  |
| T1 / RI | .761 | .194 |  |  | .645 | .229 | -.640 |  | .388 | .715 | .221 | .169 |  | -.173 | 11.866 |  |
| Change^†^ | -.027 | .137 | -1.848 | .068 | -.088 | .182 | -6.302 | .000 | 2.282 | .023 | .196 | 1.073 | .286 | .370 | .000 | 1.349 |
| T2 / RI | .748 | .219 |  |  | .666 | .229 | -.521 |  | -.044 | .763 | .213 | .326 |  | -.297 | 21.631 |  |
| Change^†^ | -.011 | .169 | -.557 | .579 | -.088 | .177 | -6.642 | .000 | 1.523 | .055 | .179 | 3.066 | .003 | .650 | .000 | 1.018 |
| T3 / RI | .814 | .209 |  |  | .634 | .243 | -.817 |  | .438 | .782 | .189 | .457 |  | -.029 | 3.320 |  |
| Change^†^ | .026 | .143 | 1.363 | .178 | -.117 | .202 | -7.092 | .000 | 1.892 | .065 | .180 | 3.373 | .001 | .611 | .000 | 1.704 |
| T4 / RI | .861 | .153 |  |  | .654 | .261 | -.550 |  | .176 | .837 | .147 | .449 |  | .054 | 19.443 |  |
| Change^†^ | .035 | .169 | 1.327 | .192 | -.100 | .247 | -4.392 | .000 | 1.659 | .082 | .140 | 5.010 | .000 | 1.638 | .000 | 2.330 |
| T5 / RI | .825 | .028 |  |  | .647 | .024 | -.685 |  | .364 | .879 | .014 | .714 |  | -.025 | 23.884 |  |
| Change^†^ | .016 | .146 | .704 | .486 | -.100 | .240 | -4.586 | .000 | 2.166 | .104 | .128 | 6.667 | .000 | .917 | .000 | 1.813 |
| T6 / RI | .819 | .187 |  |  | .677 | .270 | -.289 |  | -.203 | .893 | .132 | .495 |  | -.085 | 11.107 |  |
| Change^†^ | .037 | .197 | 1.195 | .239 | -.057 | .258 | -2.299 | .023 | .677 | .098 | .123 | 6.420 | .000 | 1.322 | .000 | .734 |
| EQ-5D-3L baseline | .835 | .159 |  |  | .798 | .166 |  |  |  | .748 | .156 |  |  |  |  |  |
| T1 / RI | .853 | .154 |  |  | .763 | .177 | -.252 |  |  | .796 | .185 | .342 |  |  | 8.795 |  |
| Change^†^ | .018 | .140 | 1.196 | .235 | -.035 | .167 | -2.762 | .006 |  | .048 | .150 | 2.902 | .005 |  | .000 |  |
| T2 / RI | .844 | .139 |  |  | .744 | .223 | -.566 |  |  | .844 | .162 | .624 |  |  | 21.240 |  |
| Change^†^ | .021 | .119 | 1.444 | .153 | -.067 | .207 | -4.362 | .000 |  | .074 | .157 | 4.716 | .000 |  | .000 |  |
| T3 / RI | .871 | .192 |  |  | .739 | .229 | -.380 |  |  | .861 | .148 | .486 |  |  | 17.790 |  |
| Change^†^ | .015 | .183 | .612 | .543 | -.069 | .227 | -3.749 | .000 |  | .089 | .149 | 5.520 | .000 |  | .000 |  |
| T4 / RI | .898 | .125 |  |  | .747 | .253 | -.374 |  |  | .861 | .154 | .394 |  |  | 8.344 |  |
| Change^†^ | .032 | .158 | 1.277 | .209 | -.059 | .242 | -2.647 | .009 |  | .062 | .175 | 3.058 | .003 |  | .000 |  |
| T5 / RI | .893 | .026 |  |  | .751 | .022 | -.321 |  |  | .924 | .013 | .739 |  |  | 13.175 |  |
| Change^†^ | .024 | .154 | .999 | .324 | -.050 | .259 | -2.117 | .036 |  | .114 | .129 | 7.270 | .000 |  | .000 |  |
| T6 / RI | .869 | .163 |  |  | .705 | .277 | -.492 |  |  | .927 | .129 | .580 |  |  | 15.140 |  |
| Change^†^ | .033 | .182 | 1.159 | .253 | -.089 | .273 | -3.396 | .001 |  | .105 | .175 | 4.856 | .000 |  | .000 |  |
| **UK** |  |  |  |  |  |  |  |  |  |  |  |  |  |  |  |  |
| QLU-C10D baseline | .800 | .169 |  |  | .751 | .178 |  |  |  | .711 | .203 |  |  |  |  |  |
| T1 / RI | .774 | .178 |  |  | .673 | .206 | -.636 |  | .376 | .730 | .202 | .153 |  | -.221 | 11.040 |  |
| Change^†^ | -.026 | .123 | -2.041 | .044 | -.078 | .164 | -6.239 | .000 | 2.688 | .019 | .179 | .957 | .341 | .388 | .000 | 1.548 |
| T2 / RI | .761 | .198 |  |  | .688 | .213 | -.522 |  | -.024 | .780 | .193 | .323 |  | -.313 | 20.971 |  |
| Change^†^ | -.014 | .156 | -.713 | .478 | -.081 | .168 | -6.482 | .000 | 1.554 | .050 | .163 | 3.079 | .003 | .760 | .000 | 1.102 |
| T3 / RI | .824 | .185 |  |  | .661 | .224 | -.820 |  | .473 | .793 | .175 | .430 |  | -.104 | 26.667 |  |
| Change^†^ | .019 | .126 | 1.117 | .269 | -.103 | .189 | -6.697 | .000 | 2.063 | .054 | .161 | 3.119 | .002 | .633 | .000 | 1.722 |
| T4 / RI | .857 | .147 |  |  | .683 | .238 | -.539 |  | .148 | .842 | .136 | .415 |  | -.012 | 16.840 |  |
| Change^†^ | .037 | .163 | 1.462 | .151 | -.088 | .229 | -4.140 | .000 | 1.540 | .067 | .126 | 4.559 | .000 | 1.489 | .000 | 2.054 |
| T5 / RI | .828 | .025 |  |  | .673 | .022 | -.705 |  | .346 | .882 | .013 | .683 |  | -.165 | 21.855 |  |
| Change^†^ | .005 | .128 | .262 | .795 | -.090 | .219 | -4.558 | .000 | 2.521 | .088 | .118 | 6.118 | .000 | .974 | .000 | 2.109 |
| T6 / RI | .828 | .172 |  |  | .698 | .252 | -.290 |  | -.235 | .900 | .116 | .477 |  | -.090 | 11.269 |  |
| Change^†^ | .037 | .186 | 1.257 | .216 | -.054 | .237 | -2.369 | .020 | .695 | .089 | .111 | 6.455 | .000 | 1.494 | .000 | .798 |
| EQ-5D-3L baseline | .814 | .224 |  |  | .756 | .245 |  |  |  | .696 | .264 |  |  |  |  |  |
| T1 / RI | .841 | .194 |  |  | .713 | .259 | -.260 |  |  | .759 | .267 | .375 |  |  | 7.131 |  |
| Change^†^ | .027 | .167 | 1.536 | .128 | -.044 | .246 | -2.321 | .021 |  | .063 | .232 | 2.464 | .016 |  | .001 |  |
| T2 / RI | .838 | .164 |  |  | .687 | .315 | -.546 |  |  | .818 | .222 | .637 |  |  | 19.024 |  |
| Change^†^ | .030 | .162 | 1.554 | .125 | -.088 | .283 | -4.172 | .000 |  | .103 | .253 | 4.051 | .000 |  | .000 |  |
| T3 / RI | .837 | .262 |  |  | .693 | .313 | -.347 |  |  | .845 | .186 | .534 |  |  | 15.484 |  |
| Change^†^ | -.007 | .233 | -.228 | .820 | -.081 | .305 | -3.246 | .001 |  | .124 | .234 | 4.926 | .000 |  | .000 |  |
| T4 / RI | .885 | .167 |  |  | .689 | .344 | -.391 |  |  | .849 | .201 | .427 |  |  | 8.198 |  |
| Change^†^ | .016 | .198 | .504 | .617 | -.077 | .312 | -2.688 | .008 |  | .085 | .236 | 3.061 | .003 |  | .000 |  |
| T5 / RI | .891 | .031 |  |  | .709 | .030 | -.358 |  |  | .920 | .014 | .848 |  |  | 1.363 |  |
| Change^†^ | .028 | .163 | 1.093 | .281 | -.058 | .357 | -1.808 | .073 |  | .138 | .181 | 6.281 | .000 |  | .000 |  |
| T6 / RI | .854 | .203 |  |  | .640 | .380 | -.525 |  |  | .924 | .159 | .567 |  |  | 14.118 |  |
| Change^†^ | .045 | .231 | 1.242 | .222 | -.121 | .369 | -3.411 | .001 |  | .131 | .245 | 4.322 | .000 |  | .000 |  |
| **USA** |  |  |  |  |  |  |  |  |  |  |  |  |  |  |  |  |
| QLU-C10D baseline | .838 | .155 |  |  | .788 | .173 |  |  |  | .746 | .197 |  |  |  |  |  |
| T1 / RI | .813 | .166 |  |  | .709 | .208 | -.652 |  | .389 | .767 | .199 | .175 |  | -.178 | 11.690 |  |
| Change^†^ | -.024 | .121 | -1.926 | .057 | -.079 | .163 | -6.328 | .000 | 2.522 | .021 | .184 | 1.042 | .300 | .414 | .000 | 1.541 |
| T2 / RI | .797 | .198 |  |  | .726 | .210 | -.500 |  | -.037 | .809 | .190 | .273 |  | -.375 | 18.209 |  |
| Change^†^ | -.011 | .158 | -.555 | .581 | -.079 | .165 | -6.370 | .000 | 1.515 | .043 | .165 | 2.586 | .011 | .613 | .000 | .909 |
| T3 / RI | .856 | .180 |  |  | .700 | .223 | -.764 |  | .391 | .829 | .160 | .429 |  | -.096 | 27.971 |  |
| Change^†^ | .015 | .133 | .863 | .392 | -.101 | .180 | -6.872 | .000 | 1.937 | .057 | .156 | 3.379 | .001 | .653 | .000 | 1.647 |
| T4 / RI | .887 | .141 |  |  | .718 | .232 | -.578 |  | .196 | .880 | .116 | .484 |  | .038 | 18.855 |  |
| Change^†^ | .027 | .153 | 1.134 | .263 | -.088 | .222 | -4.309 | .000 | 1.554 | .074 | .123 | 5.122 | .000 | 1.580 | .000 | 2.069 |
| T5 / RI | .862 | .023 |  |  | .709 | .021 | -.716 |  | .393 | .912 | .011 | .688 |  | -.128 | 22.171 |  |
| Change^†^ | .006 | .128 | .310 | .758 | -.092 | .220 | -4.599 | .000 | 2.448 | .088 | .118 | 6.156 | .000 | .919 | .000 | 1.863 |
| T6 / RI | .853 | .162 |  |  | .736 | .244 | -.294 |  | -.224 | .921 | .108 | .422 |  | -.176 | 9.392 |  |
| Change^†^ | .025 | .178 | .888 | .380 | -.052 | .231 | -2.358 | .020 | .690 | .075 | .110 | 5.516 | .000 | 1.207 | .000 | .622 |
| EQ-5D-3L baseline | .852 | .157 |  |  | .810 | .172 |  |  |  | .763 | .177 |  |  |  |  |  |
| T1 / RI | .871 | .145 |  |  | .777 | .184 | -.262 |  |  | .808 | .194 | .353 |  |  | 7.584 |  |
| Change^†^ | .019 | .127 | 1.405 | .164 | -.033 | .174 | -2.509 | .013 |  | .045 | .162 | 2.516 | .014 |  | .001 |  |
| T2 / RI | .868 | .123 |  |  | .760 | .219 | -.537 |  |  | .856 | .165 | .648 |  |  | 20.030 |  |
| Change^†^ | .024 | .117 | 1.717 | .091 | -.063 | .200 | -4.206 | .000 |  | .076 | .179 | 4.221 | .000 |  | .000 |  |
| T3 / RI | .875 | .187 |  |  | .758 | .226 | -.373 |  |  | .876 | .139 | .524 |  |  | 16.982 |  |
| Change^†^ | .005 | .171 | .215 | .831 | -.064 | .220 | -3.548 | .001 |  | .090 | .161 | 5.173 | .000 |  | .000 |  |
| T4 / RI | .904 | .127 |  |  | .763 | .241 | -.382 |  |  | .876 | .149 | .446 |  |  | 9.111 |  |
| Change^†^ | .015 | .147 | .643 | .524 | -.056 | .219 | -2.773 | .006 |  | .066 | .173 | 3.242 | .002 |  | .000 |  |
| T5 / RI | .908 | .024 |  |  | .773 | .022 | -.323 |  |  | .933 | .011 | .816 |  |  | 11.903 |  |
| Change^†^ | .020 | .133 | .977 | .334 | -.043 | .253 | -1.879 | .063 |  | .109 | .134 | 6.699 | .000 |  | .000 |  |
| T6 / RI | .883 | .150 |  |  | .725 | .272 | -.518 |  |  | .935 | .120 | .599 |  |  | 15.101 |  |
| Change^†^ | .037 | .168 | 1.416 | .164 | -.087 | .265 | -3.415 | .001 |  | .101 | .177 | 4.571 | .000 |  | .000 |  |
|  |  |  |  |  |  |  |  |  |  |  |  |  |  |  |  |  |
|  |  |  |  |  |  |  |  |  |  |  |  |  |  |  |  |  |
| * RE is calculated with t-values of t-values of paired t-tests (within group change);  ** RE is calculated with f-values of one-way ANOVA (testing for between group differences of stable, decrease and increase group mean change)  † Mean Change = Tx-score – Baseline-score;  *Abbreviations:* **t** = t-value of t-test; **F** = f-value of one-way ANOVA; **p** = p-value of t-tests, f-value; **SD** = Standard Deviation; **RI** = Responsiveness Index (= mean change within decrease OR increase group / SD stable group); **DRI** = Difference RI (QLU-C10D - EQ-5D-3L); **RE** = Relative Efficiency; Betw. = between. | | | | | | | | | | | | | | | | |

| APPENDIX D: Results Responsiveness Analysis – response groups (progressive vs. non-progressive) | | | | | | | | | | | | | | | | | |
| --- | --- | --- | --- | --- | --- | --- | --- | --- | --- | --- | --- | --- | --- | --- | --- | --- | --- |
|  | progressive^†^ | | | | non-progress. | | | |  |  | **RE** | Between groups | | | | | |
| Response | Base | Mean *change‡* | SD | t* | Base | Mean *change‡* | SD | t* | **RI** | **DRI** | Progr. | **t*** | **Mean** diff.‡ | **SD** pooled | **Cohen’s d** | **Diff.** Cohen’s | **RE** |
|  |  |  |  |  |  |  |  |  |  |  | *Non-prgr.* |  |  |  |  |  |  |
| *Baseline – T5* | n = 32^†^ QLU-C10D p ≤ 0.01 | | | | n = 202 p ≥ 0.21 | | | |  |  |  | N = 234  QLU-C10D p ≤ 0.01 | | |  |  |  |
| AUS QLU-C10D | .789 | -.116 | .240 | **-2.722** | .783 | -.009 | .202 | -.626 | -.573 | .337 | 2.890 | **-2.705** | -.107 | .207 | -.515 [-.890; -.139] | .228 | 1.796  [1.643; 1.949] |
| AUS EQ5D | .759 | -.057 | .342 | -.942 | .799 | .017 | .242 | .986 | -.236 |  |  | -1.506 | -.074 | .258 | -.287 [-.660; .087] |  |  |
| CAN QLU-C10D | .773 | -.120 | .248 | **-2.732** | .766 | -.008 | .212 | -.543 | -.564 | .281 | 2.782 | **-2.698** | -.111 | .217 | -.513 [-.889; -.138] | .177 | 1.525  [1.410; 1.640] |
| CAN EQ5D | .788 | -.057 | .330 | -.982 | .822 | .018 | .202 | 1.263 | -.283 |  |  | -1.769 | -.075 | .224 | -.337 [-.711; .038] |  |  |
| UK QLU-C10D | .789 | -.116 | .231 | **-2.843** | .782 | -.010 | .188 | -.726 | -.617 | .424 | 3.837 | **-2.879** | -.107 | .195 | -.548 [-.924; -.172] | .304 | 2.246  [2.031; 2.460] |
| UK EQ5D | .746 | -.054 | .415 | -.741 | .797 | .019 | .280 | .980 | -.194 |  |  | -1.282 | -.074 | .302 | -.244 [-.618; .130] |  |  |
| US QLU-C10D | .822 | -.116 | .238 | **-2.753** | .817 | -.010 | .189 | -.774 | -.611 | .421 | 3.792 | **-2.818** | -.105 | .197 | -.536 [-.912; -.160] | .287 | 2.150  [1.948; 2.351] |
| US EQ5D | .799 | -.039 | .301 | -.726 | .837 | .016 | .203 | 1.114 | -.190 |  |  | -1.311 | -.055 | .219 | -.249 [-.623; .124] |  |  |
| ^†^ Median days since progression: T3 = 58, T4 = 116, T5 121, T6 = 170;  * **bold** t-test values signify p-values ≤ .01;  ‡ Mean Change = T_x_-score – Baseline-score; Mean Difference = Non-progressive – Progressive;  **Abbreviations:** t = t-value; SD = Standard Deviation; RI = Responsiveness Index; DRI = Difference (QLU-C10D – EQ-5D-3L) of RI; RE = Relative Efficiency (Quotient t-values: QLU-C10D / EQ-5D-3L); Diff. Cohen’s = Difference (QLU-C10D – EQ-5D-3L) of Cohen’s d. | | | | | | | | | | | | | | | | | |

Appendix E: Bias Analysis

| Sample Characteristics | Orig.data | | Selected sample | | | | | | | | | | | | | |
| --- | --- | --- | --- | --- | --- | --- | --- | --- | --- | --- | --- | --- | --- | --- | --- | --- |
|  |  |  | T0 | | T1 | | T2 | | T3 | | T4 | | T5 | | T6 | |
| N | 545 | | 435 | | 329 | | 313 | | 269 | | 223 | | 219 | | 192 | |
|  | n | % | n | % | n | % | n | % | n | % | n | % | n | % | n | % |
| Age (mean) / (SD); (median) | 56.5 / 11 | 57.94 | 56,2 / 11 | 57.9 | 55.8/11 | 57.5 | 55.8 / 11 | 57.5 | 54.6 / 12 | 56.8 | 53.4 / 11 | 54.2 | 53.6 / 12 | 54.7 | 53.6 / 11 | 54.8 |
| Sex male | 291 | 53.4 | 236 | 54.3 | 183 | 55.6 | 172 | 55.0 | 151 | 56.1 | 123 | 55.2 | 126 | 57.5 | 111 | 57.8 |
| female | 254 | 46.6 | 199 | 45.7 | 146 | 44.4 | 141 | 45.0 | 118 | 43.9 | 100 | 44.8 | 93 | 42.5 | 81 | 42.2 |
| Overall survival status: alive or LFI | 263 | 48.3 | 217 | 49.9 | 170 | 51.7 | 175 | 55.9 | 150 | 55.8 | 142 | 63.7 | 139 | 63.5 | 134 | 69.8 |
| dead | 282 | 51.7 | 218 | 50.1 | 159 | 48.3 | 138 | 44.1 | 119 | 44.2 | 81 | 36.3 | 80 | 36.5 | 58 | 30.2 |
| MMSE:  < 27 | 106 | 19.4 | 83 | 19.1 | 52 | 15.8 | 47 | 15.0 | 42 | 15.6 | 36 | 16.1 | 33 | 15.1 | 33 | 17.2 |
| ≥ 27 | 432 | 79.3 | 352 | 80.9 | 277 | 84.2 | 266 | 85.0 | 227 | 84.4 | 187 | 83.9 | 186 | 84.9 | 159 | 82.8 |
| Progression free:  non progressive | 158 | 29.0 | 123 | 28.3 | 91 | 27.7 | 79 | 25.2 | 74 | 27.5 | 63 | 28.3 | 67 | 30.6 | 60 | 31.3 |
| Progressive / dead | 387 | 71.0 | 312 | 71.7 | 238 | 72.3 | 234 | 74.8 | 195 | 72.5 | 160 | 71.7 | 152 | 69.4 | 132 | 68.8 |
| Steroid at baseline  No | 329 | 60.4 | 268 | 61.6 | 203 | 61.7 | 202 | 64.5 | 181 | 67.3 | 150 | 67.3 | 138 | 63.0 | 124 | 64.6 |
| Yes | 216 | 39.6 | 167 | 38.4 | 126 | 38.3 | 111 | 35.5 | 88 | 32.7 | 73 | 32.7 | 81 | 37.0 | 68 | 35.4 |
| Treatm.:  Chemoradiotherapy | 273 | 50.1 | 218 | 50.1 | 167 | 50.8 | 162 | 51.8 | 135 | 50.2 | 112 | 50.2 | 109 | 49.8 | 96 | 50.0 |
| Added Cilengitide | 272 | 49.9 | 217 | 49.9 | 162 | 49.2 | 151 | 48.2 | 134 | 49.8 | 111 | 49.8 | 110 | 50.2 | 96 | 50-0 |
| WHO  Performance status 0 | 309 | 56.7 | 252 | 57.9 | 195 | 59.3 | 194 | 62.0 | 170 | 63.2 | 142 | 63.7 | 136 | 62.1 | 117 | 60.9 |
| PS > 0 | 236 | 43.3 | 183 | 42.1 | 134 | 40.7 | 119 | 38.0 | 99 | 36.8 | 81 | 36.3 | 83 | 37.9 | 75 | 39.1 |
| Extent of surgery:  Biopsy | 16 | 2.9 | 14 | 3.2 | 9 | 2.8 | 8 | 2.6 | 8 | 3.0 | 7 | 3.1 | 6 | 2.7 | 5 | 2.6 |
| Partial resection | 258 | 47.3 | 211 | 48.5 | 165 | 50.4 | 148 | 47.3 | 135 | 50.6 | 102 | 45.7 | 107 | 48.9 | 82 | 42.9 |
| Complete resection | 269 | 49.4 | 208 | 47.8 | 153 | 46.8 | 155 | 49.5 | 124 | 46.4 | 113 | 50.7 | 106 | 48.4 | 104 | 54.5 |
| Overall survival  (days): (median); (SD) | 706 | 323.8 | 710 | 318.6 | 707 | 304.3 | 744 | 289.3 | 748 | 272.2 | 774 | 259.7 | 787 | 254.7 | 816 | 225.4 |
| Progression free survival: days (median); (SD) | 246 | 312 | 255 | 310.3 | 322 | 308.6 | 400 | 307.9 | 413 | 291.8 | 510 | 289.7 | 568 | 285.1 | 598 | 268.3 |

| Appendix E: Bias Analysis | | | | | | | |  |
| --- | --- | --- | --- | --- | --- | --- | --- | --- |
|  | V4 | V30 | V100 | V180 | V260 | V340 | V500 |  |
|  | **T0** | **T1** | **T2** | **T3** | **T4** | **T5** | **T6** |  |
| **N (orig. Study Data)** | 545 | 442 | 409 | 347 | 293 | 291 | 262 |  |
| **N (selected)** | 435 | 348 | 346 | 295 | 233 | 234 | 215 |  |
| ***Difference*** | *119* | *94* | *63* | *52* | *60* | *57* | *47* |  |
| ***%*** | *21.48* | *21.27* | *15.40* | *14.99* | *20.48* | *19.59* | *17.94* |  |
|  |  |  |  |  |  |  |  |  |
| **CHI^2^-Values** |  |  |  |  |  |  |  |  |
| 1) Sex (male. female) | 0.245 | 1.064 | 0.163 | 0.13 | 0.417 | 1.746 | 0.092 |  |
| 2) Randomization Group (chemoradiotherapy & temozolomide vs. added Cilengitide) | 0.000 | 0.019 | 1.426 | 0.109 | 1.417 | 0.9790 | 2.058 |  |
| 3) Overall Survival (alive vs. dead) | 2.017 | 0.093 | 3.649 | 0.036 | 2.245 | 0.978 | 2.231 |  |
| 4) Progr.-free Survival (non-progressive, progressive) | 0.362 | 0.032 | 0.933 | 0.002 | 0.014 | 0.205 | 0.466 |  |
| 5) WHO Performance status (0 vs. > 0) | 0.019 | 0.072 | 0.411 | 0.019 | 0.042 | 1.267 | 3.168 |  |
| 6) MMSE | 0.607 | 2.462 | 2.942 | 0.04 | 0.116 | 0.215 | 1.026 |  |
| 7) Steroid at baseline (no, yes) | **4.641*** | 1.038 | 4.055 | **6.103*** | **6.209*** | 0.503 | 3.734 |  |
| 8) Extent of Surgery^c^ | 0.346 | 1.559 | 0.937 | 2.473 | 0.91 | 1.56 | 0.707 |  |
|  |  |  |  |  |  |  |  |  |
| **T-Test^b^** |  |  |  |  |  |  |  |  |
| Age | n.s. | n.s. | n.s. | n.s. | 0.014 | 0.04 | 0.035 |  |
| Progression | n.s. | n.s. | n.s. | n.s. | n.s. | n.s. | n.s. |  |
| Overall Survival | n.s. | n.s. | 0.027 | n.s. | n.s. | n.s. | n.s. |  |
|  |  |  |  |  |  |  |  |  |
| a Exclusion criteria: missing clinical data, missing or non-plausible dates of data collection, missing QoL data;  b n.s. = not significant (> .05): this result indicates that no significant differences between the selected and the total sample was detected with respect to the tested variable; in every other case the p-value of the t-test is reported;  c Chi-squared test with three groups: total resection, partial resection, biopsy; biopsy had less than 5 expected cases, thus Fisher’s tests significance values are used in this row. | | | | | | | | |
